# Thiel embalming in neonates: methodology and benefits in medical training

**DOI:** 10.1007/s12565-022-00650-1

**Published:** 2022-02-08

**Authors:** Francisco Sanchez-Ferrer, Maria Dolores Grima-Murcia, Francisco Sánchez-del-Campo, Maria Luisa Sánchez-Ferrer, Eduardo Fernández-Jover

**Affiliations:** 1 Medical School, University of Miguel Hernández de Elche, Sant Joan d’Alacant, Spain; 2 Anatomical Innovation Service, University of Miguel Hernández de Elche, Sant Joan d’Alacant, Spain; 3 Medical School, University of Murcia, Murcia, Spain; 4 Bioengineering Institute, University of Miguel Hernández de Elche, CIBER BBN, Elche, Spain; 5 Pediatric Service, Pediatric Cardiology Unit, San Juan de Alicante University Hospital (Alicante, Spain), Carretera Nacional 332, Alicante-Valencia s/n, 03550 Sant Joan d’Alacant, Spain

**Keywords:** Donation, Thiel embalming, Cadavers, Pediatrics, Medical training

## Abstract

Current teaching and training methods for surgical techniques in the pediatric population involve artificial models (manikins), animals or adult human cadavers embalmed using various techniques. We found no references in the literature concerning the use of the Thiel method in the pediatric population. The aim of this study, therefore, was to assess the viability of using pediatric human cadavers embalmed through Thiel’s technique and to compare them with standard pediatric manikins. After donation of a 24-week stillborn, the Thiel technique was carried out for fixation following the usual protocol. A video recording with eye-tracking glasses was used to perform an examination, and techniques. The same procedures were conducted on a pediatric manikin. Medical students, medical residents and physicians were asked to respond to questions in an online survey after being shown the video. A total of 92 responses were obtained. The Thiel-embalmed stillborn was assessed as superior to the manikin in all items. Our study confirmed that this technique is feasible even with extremely small donors. The value of this form of preservation for medical training is not widely known though it is receiving increasing interest. Our results show that Thiel fixation in pediatrics is clearly more highly valued than a manikin and offers great potential. This innovative application of the Thiel method in the pediatric population is technically possible. It poses no additional difficulties and is very positively assessed for undergraduate and postgraduate teaching.

**Supplementary Information:**

The online version contains supplementary material available at 10.1007/s12565-022-00650-1.

## Introduction

Human cadavers are not only used for the study of human anatomy. They also play an important role as anatomical models in situations where it is impractical, illegal, or unethical to work with patients, as is the case with surgical specialty training or intensive care unit techniques (Jaung et al. 2011; Groscurth et al. 2001). The use of cadavers is also helpful in the preclinical development of instruments and procedures, enabling products to be tested safely without risk to patients but with a high degree of similarity.

Although other models such as augmented or virtual reality (Kugelmann et al. 2018), 3D printed models, animals or manikins are also used, it is difficult to achieve the degree of realism offered by the human body. The bodies donated for scientific and training purposes, generously bequeathed by donors, are known as “silent teachers” (Eisma and Wilkinson 2014).

The history of techniques to preserve the human body goes back many centuries and includes natural means such as mummification or freezing and artificial methods such as immersion or arterial injection (Brenner 2014). Embalmed cadavers are employed in education, training, and research with minimal risk of infection, and they allow for prolonged use. Nonetheless, depending on the embalming method, changes in color, mobility, or tissue texture occur (Brenner 2014). Fresh-frozen bodies are more realistic and flexible, but the time available before deterioration is short and the risk of infection for the user increases. For many years, the most widespread technique worldwide for embalming has involved the use of formalin. Formalin-embalmed cadavers have great durability but are not very useful for surgical training, such as laparoscopy.

An embalming method named after its discoverer, Thiel, has been in use since the 1990s (Thiel 1992b, 2002). Through this form of preservation, the cadavers remain flexible, with coloring, mobility and texture very similar to a living patient. This method also enables preservation (including the internal organs) over a long period of time and is ideal for many activities including examination techniques (Feigl et al. 2020; Aurore et al. 2020), radiological tests and surgery (Odobescu et al. 2014; Benkhadra et al. 2009; Giger et al. 2008). This type of embalming is still relatively unknown and is used in very few places in Europe, possibly because the original text was published in German (Benkhadra et al. 2011).

With respect to training in pediatric patients, both the degree and the specialization are based on references in books, virtual reality, pediatric simulation manikins or clinical practice itself when possible (Harada et al. 2015; Stone et al. 2014; Trudeau et al. 2018; Lopreiato and Sawyer 2015). Indeed, much of this training must be performed on adult human references such as cadavers embalmed using different techniques and found in the anatomy laboratories (Prigge et al. 2014; Wagner et al. 2018).

This study examines the potential use of Thiel embalming for pediatric cadavers donated to science. We evaluate its usefulness and the opinions of students and professionals regarding the results.

## Materials and methods

### Technique and procedures

Embalming was performed using Thiel’s technique on a fetus deceased in utero at 24 weeks of gestation, weighing 680 g, donated to the Department of Anatomy and Histology of Miguel Hernández University in 2015. The donation was carried out according to the legislation in force at the time.

Once the cadaver was received in the department, the infusion was performed by cannulating the umbilical vessels with the different fluids used in Thiel’s technique, which include monopropylene glycol, ammonium nitrate, potassium nitrate, sodium sulfite, boric acid, chlorocresol, formaldehyde, alcohol and morphine (Thiel 1992a, b, 2002; Eisma and Wilkinson 2014). Subsequently, the cadaver was preserved in embalming fluid for 6 months.

### Video recording and comparison between Thiel cadaver and neonatal manikin

A pediatrician evaluated the different techniques for examination, mobility, ultrasound, lumbar puncture and intubation as well as the general characteristics of the neonate preserved using Thiel’s method. The same procedures were carried out on a neonatal manikin.

All these actions were video recorded (using both the Thiel-fixed neonatal cadaver and the manikin) through eye-tracking glasses (Tobii Glasses 2), providing a first-person perspective. Throughout the video, a circle can be seen, which indicates where the physician’s gaze is focused at all times (see Video).

The TitanR (SonoSite) ultrasound machine with a high frequency linear probe was used for the ultrasound assessment.

### Survey for students and professionals

A voluntary, anonymous online questionnaire was administered for evaluation of the video by medical students, medical residents and attending physicians (Annex 1).

The participants first answered questions regarding demographic information then questions about their comparison of the degree of realism of the Thiel-cadaver and the manikin as well as their assessment of teaching opportunities and which techniques provided the greatest potential. A scale of 1–5 (1 very poor–5 very good) was used for the evaluation of both training options.

## Results

The techniques and procedures for preserving neonatal cadavers are similar to those used for adult cadavers, except that it is more difficult to cannulate the femoral or jugular vein in the neonate, while the umbilical vein is more accessible. We, therefore, cannulated the umbilical vein with a neonatal catheter. The solution used was the same as that routinely used for infusion in adult cadavers.

The results in terms of mobility and texture were comparable to those of an adult cadaver, although in our case the skin color was patchy. The internal organs visualized by ultrasound allowed us to see their basic anatomy. The visualization of the glottis for intubation allowed us to observe the perfect conservation of the oropharynx and glottis.

### Survey

The questionnaire was administered between July and September 2019 and 92 people responded, of whom 77.2% were women and 22.8% were men. There was an even distribution across the three target groups with 29.3% students, 37% medical residents and 33.7% attending physicians.

**Fig. 1:**
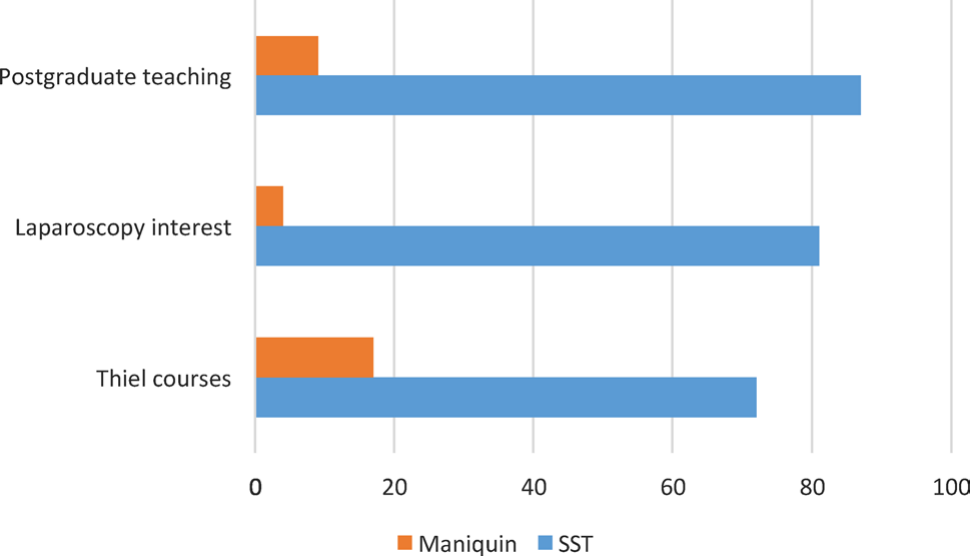
The percentage of positive or negative responses to interest in Thiel courses, interest in the Thiel method for laparoscopy or general interest for postgraduate teaching

Responses concerning whether respondents are interested in Thiel courses, how the Thiel method is of interest in laparoscopy and whether they believe that postgraduate teaching is of interest are shown in Fig. 1.

After viewing the video, the responses concerning the Thiel-fixed neonatal cadaver (SST) were significantly higher than for the manikin (Table 1).

**Table 1:** Comparison between Thiel-fixed cadaver (SST) and neonatal manikin

*N* = 92 (%)	Thiel (SST)	Manikin
Lifelike	84 (91%)	7 (7.6%)
Teaching opportunities	66 (71.7%)	23 (25%)

**Fig. 2:**
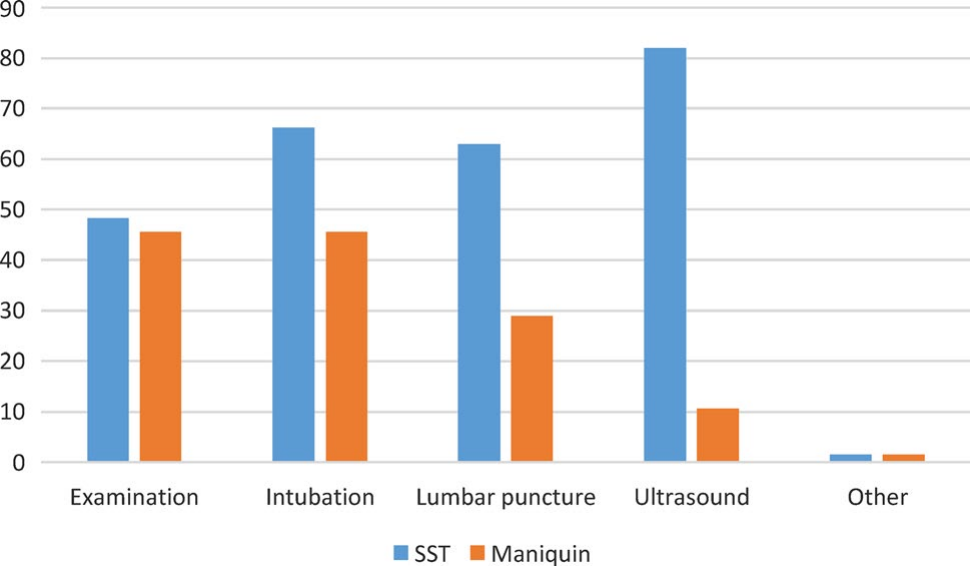
Survey responses on the usefulness of the Thiel cadaver (SST) and the manikin in different procedures

Figure 2 depicts the comparison between techniques. In no case was the manikin considered superior to the SST, with the differences being much greater for ultrasound and lumbar puncture.

The scores on the scale from 1 to 5 showed a mean value for the SST of 4.37 and for the manikin of 3.43 (*p* < 0.001). The comments provided at the end of the survey revealed considerable interest in Thiel’s fixation technique and, in many cases, surprise at the lack of knowledge about it.

## Discussion

The use of Thiel embalming is growing due to the applications and opportunities it provides (Boaz et al. 2013, no date). An increasing number of healthcare professionals are using adult cadavers fixed with this method for the study of anatomy, learning techniques, professional training and for testing materials in preclinical research as these activities can present risks in real patients (Boaz and Anderhuber 2009, no date). Thiel-embalmed donor animals are also increasingly used for veterinary training (Nam et al. 2020) as well as in medical training when human donors are scarce.

Since 2016, the occupational exposure limit to formaldehyde was lowered by the European Committee on Hazardous Substances, as formaldehyde had been classified as a human 1B carcinogen. For this reason, in 2018 the Anatomische Gesellschaft working group issued recommendations to limit exposure to formaldehyde in anatomy laboratories (Waschke et al. 2019). The Thiel method on the other hand has no proven harmful effects. It is also safe at the microbiological level (Rowe et al. 2020).

Various specialties use Thiel-embalmed cadavers for training and learning purposes (Esposito et al. 2015; Hölzle et al. 2012; Wolff et al. 2008; Hassan et al. 2015; Peuker et al. 2001), particularly in laparoscopy (Giger et al. 2008) (at times combined with the use of ultrasound) (Veys et al. 2020), microsurgery (Odobescu et al. 2019), plastic surgery and neurosurgery in combination with nuclear magnetic resonance (Eljamel et al. 2014). These cadavers are also valuable in the management of the airway where the superiority of Thiel-embalmed cadavers over manikins has been demonstrated (Szucs et al. 2016).

Pediatric patients have a different anatomy and characteristics than adults. For this reason, pediatric models are especially important (Ade-Ajayi et al. 2013). A child is not a “small adult” and the differences are greater at a younger age and size. Techniques such as laparoscopy (Ade-Ajayi et al. 2013), placement of a central catheter (Thomas et al. 2013) or neonatal and pediatric intubation (O’Shea et al. 2020; Ernst et al. 2014) are all performed in pediatric training. Many of these techniques are learned with simulators, high-fidelity pediatric manikins (Azzie et al. 2011) or 3D models (Rose et al. 2015) and, in certain cases, on real patients.

Our study presents a smaller human cadaver embalmed using Thiel’s method, as described in the references. We were able to verify that this technique is feasible and that the results are similar to those of adult cadavers fixed with this technique (Eisma and Wilkinson 2014), hence the name Small Silent Teacher. The only difference in the fixation is that we obtain access for the infusion through the umbilical vessels due to the greater ease.

Thiel cadavers and our neonatal cadaver in particular present characteristics of color, texture, flexibility, mobility and internal organ preservation very similar to the living body. This has been proven in other Thiel-fixed cadavers through radiological tests such as nuclear magnetic resonance, plain-film radiology or ultrasound (Joy et al. 2015; Schramek et al. 2013). The lifelike properties of the specimens are highlighted in the publications involving the Thiel-embalming technique (Hohmann et al. 2019; Hammer et al. 2015).

Medical students and professionals assessed the neonatal Thiel-fixed cadaver very favorably and found it to be superior to the pediatric manikin with which it was compared. Those surveyed highlighted the realistic quality, the teaching opportunities it offers and its particular usefulness for techniques such as ultrasound, lumbar puncture or intubation. A single cadaver can be used for multiple consecutive procedures, even over several years, which makes its use efficient. Also, unlike formaldehyde, Thiel embalming is a safer form of preservation (Waschke et al. 2019).

We can affirm that Thiel’s embalming method is technically possible and uncomplicated in pediatric patients, offering teaching and research opportunities similar to those offered by adult Thiel cadavers. The limitations we observed are the reduced number of donated bodies in the pediatric population, firstly because of the low mortality rate and in many cases because of the lack of knowledge about the option of donation, which in many stillbirths would be possible.

## Conclusions

The novel use of Thiel’s embalming technique in neonates provides learning and training opportunities for students and physicians working with pediatric populations. Human anatomy changes with age and this method offers a combination of a high level of fidelity to the living child, safety in its use and preservation of the body.

### Electronic supplementary material

Below is the link to the electronic supplementary material.

Supplementary file1 (MP4 10164 KB)

## Annex

Link to Video: https://www.youtube.com/watch?reload=9&time_continue=73&v=lpxePxiMNBs&feature=emb_logo

Link to Survey: https://docs.google.com/forms/d/e/1FAIpQLScXJgrIvxX_wPa9s8KPelpSirIFqPL-j12liH1f5G-5R7keqw/viewform?vc=0&c=0&w=1
